# The Roles of Co-Chaperone CCRP/DNAJC7 in *Cyp2b10* Gene Activation and Steatosis Development in Mouse Livers

**DOI:** 10.1371/journal.pone.0115663

**Published:** 2014-12-26

**Authors:** Marumi Ohno, Tomohiko Kanayama, Rick Moore, Manas Ray, Masahiko Negishi

**Affiliations:** 1 Pharmacogenetics Section, Reproductive and Developmental Biology Laboratory, National Institute of Environmental Health Sciences, National Institutes of Health, Research Triangle Park, North Carolina, United States of America; 2 Knockout Core, National Institute of Environmental Health Sciences, National Institutes of Health, Research Triangle Park, North Carolina, United States of America; Univeristy of California Riverside, United States of America

## Abstract

Cytoplasmic constitutive active/androstane receptor (CAR) retention protein (CCRP and also known as DNAJC7) is a co-chaperone previously characterized to retain nuclear receptor CAR in the cytoplasm of HepG2 cells. Here we have produced CCRP knockout (KO) mice and demonstrated that CCRP regulates CAR at multiple steps in activation of the *cytochrome* (*Cyp*) *2b10* gene in liver: nuclear accumulation, RNA polymerase II recruitment and epigenetic modifications. Phenobarbital treatment greatly increased nuclear CAR accumulation in the livers of KO males as compared to those of wild type (WT) males. Despite this accumulation, phenobarbital-induced activation of the *Cyp2b10* gene was significantly attenuated. In ChIP assays, a CAR/retinoid X receptor-α (RXRα) heterodimer binding to the *Cyp2b10* promoter was already increased before phenobarbital treatment and further pronounced after treatment. However, RNA polymerase II was barely recruited to the promoter even after phenobarbital treatment. Histone H3K27 on the *Cyp2b10* promoter was de-methylated only after phenobarbital treatment in WT but was fully de-methylated before treatment in KO males. Thus, CCRP confers phenobarbital-induced de-methylation capability to the promoter as well as the phenobarbital responsiveness of recruiting RNA polymerase II, but is not responsible for the binding between CAR and its cognate sequence, phenobarbital responsive element module. In addition, KO males developed steatotic livers and increased serum levels of total cholesterol and high density lipoprotein in response to fasting. CCRP appears to be involved in various hepatic regulations far beyond CAR-mediated drug metabolism.

## Introduction

Constitutive active receptor (CAR) was originally characterized as a drug-activated nuclear receptor that induces hepatic drug metabolism and secretion by activating genes that encode enzymes such as cytochrome P450s (CYP), sulfotransferases and UDP-glucuronosyltransferases as well as drug transporter genes [1]–[7]. Subsequently, regulation by CAR has been extended far beyond drug metabolism to hepatic energy metabolism and cell growth and death, thereby becoming a critical factor in the development of diseases including diabetes and hepatocellular carcinoma [8], [9]. Therefore, understanding the molecular mechanism of CAR activation is essential for us to predict and control both beneficial and adverse effects caused by this activation.

CAR is sequestered in its inactive form in the cytoplasm by phosphorylating its residue threonine 38; only non-phosphorylated CAR translocates into the nucleus, forms a heterodimer with RXR and activates target genes [10], [11]. Threonine 38 is phosphorylated when epidermal growth factor receptor (EGFR) signaling is stimulated, while repression of this signaling results in dephosphorylation that activates CAR [12], [13]. Consequently, CAR is, in principle, a cell signal-regulated nuclear receptor and this signal-mediated mechanism is now demonstrated in both mouse and human liver cells [13], [14]. As to regulation of CAR by therapeutic drugs, phenobarbital (PB) antagonizes EGFR signaling to dephosphorylate and activate CAR [13], while metformin represses CAR activation by preventing dephosphorylation [14]. Thus, phosphorylation of threonine 38 is an essential factor that regulates CAR activation and nuclear translocation.

In addition to this phosphorylation, we previously identified a tetratricopeptide repeat protein (TPR) that interacts with CAR to regulate its cytoplasmic localization in HepG2 cells and named this TPR protein Cytoplasmic CAR Retention Protein (CCRP) [15]. CCRP, also known as DNAJC7, is a member of the co-chaperone HSP40 family, which are structurally featured by J-domain and repeats of TPR motif, the 34-residue peptide forming a pair of anti-parallel α helices. The J-domain regulates ATP hydrolysis by HSP70, while the TPR motif mediates formation of homo-dimer or an array of hetero-complexes with non-TPR proteins via TPR motifs: co-chaperones with HSP90 and HSP70 [16]–[20]. In fact, CCRP formed a complex with CAR and HSP90, thereby causing accumulation of CAR in the cytoplasm of HepG2 cells [15]. In addition to CAR, CCRP was also found to interact with the glucocorticoid receptor (GR) and regulated its trans-activation activity in N2A and Hela cells [20]. Therefore, CCRP might be a common co-chaperone that regulates not only intracellular localization but also trans-activation activity of many nuclear receptors. However, these regulations by CCRP have not been investigated in organs and tissues such as liver *in vivo*.

Here we generated CCRP KO mice and utilized them to examine the *in vivo* roles of CCRP in CAR activation in the livers. CCRP KO mice were treated with PB, from the livers of which samples were prepared for Western blot, real time PCR, cDNA microarray and chromatin immunoprecipitation (ChIP) assays. Demonstrating that CCRP regulates not only intracellular localization of CAR but also its ability to activate the *Cyp2b10* gene, we will develop the hypothesis that CCRP determines both CAR-dependent and -independent gene expression in the livers.

## Materials and Methods

### Generation of the CCRP knockout mice

**Figure 1:**
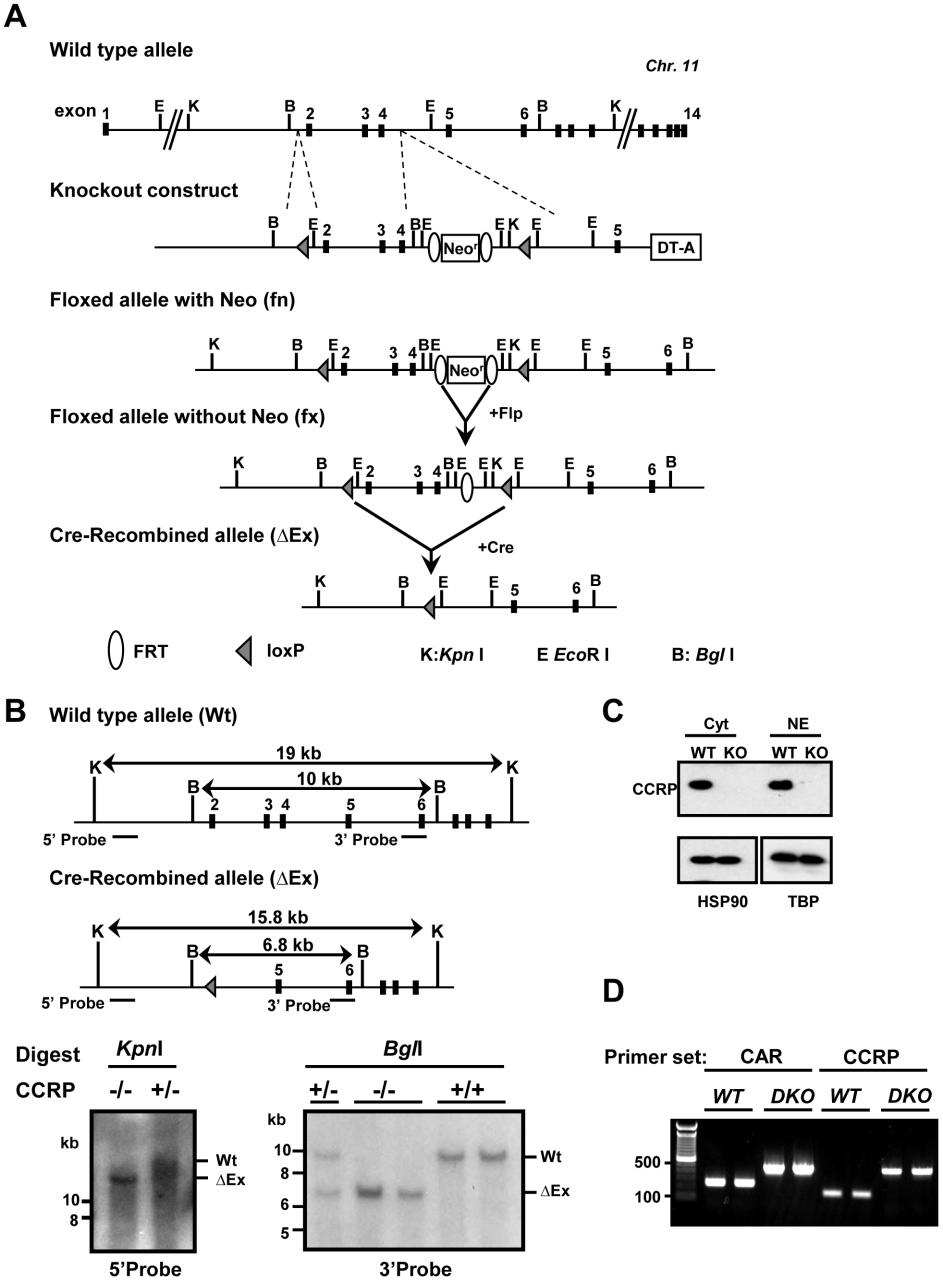
Gene targeting and conditional deletion of exon 2 to 4 of the mouse *Ccrp* gene. (A) Restriction maps of the wild type allele and knockout construct. After Cre recombination, *Ccrp* gene exons 2–4 deleted allele (ΔEx) was generated as described in material and methods. (B) Determination and confirmation of genotype for CCRP KO mice by Southern blot analyses. Expected size of digested fragments was shown. (left) *Kpn*I digested, and (right) *Bgl*I digested genomic DNA derived from CCRP KO mice were detected by indicated probes. (C) CCRP protein expression in the cytoplasm and the nuclei. The cytoplasm and nuclear proteins were extracted from the livers of WT and KO males and 10 µg proteins were subjected to western blotting as described in materials and method section. HSP90 and TBP were used as loading controls of the cytoplasm and the nuclei, respectively. (D) Genotyping result of DKO mice. Genomic DNA was extracted from ear tissue. CAR primer set generated 266 bp and 466 bp PCR products for WT and KO, respectively. CCRP primer set generated 150 bp and 450 bp products for WT and KO, respectively.

A colony of CCRP global knockout B6; 129-Dnajc7< tm1Neg > (CCRP^-/-^ or KO in this manuscript) was established at the facilities of Knockout core of NIEHS. To construct CCRP targeting vector, three fragments of the mouse *Ccrp* gene were amplified from 129SvEv genomic DNA with Pfu Turbo DNA polymerase (Promega, Madison, WI, USA); a 3.3-kb fragment containing intron 1 (left arm), a deleting 3.2-kb fragment containing exons 2-4 and a 5.4-kb fragment containing introns 4 and 5 and exon 5 (right arm). The primer sets used for these amplifications were; left arm, 5'-AAGGCGCGCCTAGGACAGATTCTTCACACCA-3', 5'-ATATGGCCGGCCTCAGGGGTGAGCTCAGAAGC-3'; deleting fragment; 5'-AGATCGATGGTATAGTGATGTGACTCTCCT-3', 5'-CTTAATTAAGCCCTCAGGGCACCTCTCATAGGAACAGATCAGACCT-3'; right arm, 5'-GCGGCCGCTGGTGCGGTGGGTCTGGAAAGAAGAG-3', 5'-GCGATCGCAGAACAAAGCAAGGCTCTAG-3'. After amplification, these fragments were digested with restriction enzymes and ligated into the corresponding sites of a Multiple Amplicon Insertion Knock Out vector which carries three multi cloning sites, *FRT-Pgk-neo-FRT* cassette, DT-A cassette, and two loxP sites (Fig. 1-A, upper part) [21]. The left arm was placed upstream of the 5′-loxp site; the deleting fragment between the 5′-loxP and the *Neo* cassette; the right arm downstream of the 3′-loxP site. The targeting vector linearized by *Sal*I was electroporated to ES cells derived from 129SvEv blastocysts. The G418-resistant ES clones were screened by Southern blot analysis; these Southern probes were PCR amplified from ES cell genome DNA using following primers. 5'-ACTGAAATCTGCATTTGCTAACGC-3' and 5'-TTGACACTTCATCCTGCAGTCTCTT-3' for 5'-probe and for 3'-probe, 5'-AGGTAGTGATATTACTTACTAACTC-3' and 5'-CTAAATTCAGTCCCCAAAACCTTCTG-3'. The targeted ES cell clones were injected into blastocysts from C57BL/6 albino mice. The obtained CCRP fx/fx male mice were bred with Sox2-Cre female mice (The Jackson Laboratory, Bar Harbor, ME, USA) to delete exons (exons 2–4) to generate a recombined null allele (ΔE/+). After intercrossing with the heterozygote mice, we obtained *CCRP^-/-^* (KO) and *CCRP* ^+/+^ (WT) mice. A CAR/CCRP double knockout mouse line was established by crossing *CCRP^-/-^* (KO) with *CAR* ^-/-^ [6]. We confirmed double knockout of CAR and CCRP genes in DKO animals by genotyping using following primer sets. For CAR; 5′-GGAGGTGTACCAGTGTTTAAAGGTGG-3′, 5′-GGGTGGGATTAGATAAATGCCTGCTCT-3′, and 5′-CCCACATTCAGGAGACCATGACAGC-3′. For CCRP; 5′- TGGCGGCATGTCGTAGTTT-3′, 5′-GTCTCAGCTTCTCCAAACTCTG-3′, and 5′-GTCCTGCCTTATTGGTCACTCTTC-3′. The PCR protocol was 95°C x 2 min; 95°C x 10 s, 58°C x 10 s, and 72°C x 30 s for 40 cycles with 50 ng of genomic DNA extracted from ear tissue in a 20 µl reaction using GreenTaq (Promega). The product size was determined by gel electrophoresis (Fig. 1-D). All animal procedures were approved by the Animal Ethics Committee of National Institute of Environmental Health Sciences.

### Southern Blots

Southern blot analyses were performed according to the manufacturer protocol. Probe labeling with [α^32^P]-dCTP were performed with using Prime-It RmT Random Primer Labeling Kit (Stratagene, La Jolla, CA, USA). And denatured labeled probe was incubated with each blot under 10^6^ cpm/ml at 42°C overnight. The positions of probes are shown as horizontal bars in Fig. 1-B. The same probes used for ES cell screening were utilized for this Southern analysis.

### Animal treatment

Animals were fed a standard chow diet and allowed to drink water ad libitum. Experiments were done on 7–10 weeks old male mice. After fasting for 24 h, animals were treated with vehicle PBS or PB at a dose of 100 mg/kg body weight for 6 h (RT-PCR, microarray, and western blot) or for 3 h (ChIP assay). After treatment, mice were sacrificed by cervical dislocation and liver samples were frozen by liquid nitrogen and stored at -80°C before use. For cholesterol and lipid study, mice were simply fasted for 24 h. After sacrifice by carbon dioxide, liver or blood samples were collected.

### Western blots

Nuclei were prepared and lysed as previously published [22] with following minor modifications; homogenizing buffer contained 2 M sucrose and no protease inhibitor. Whole liver extracts were prepared by homogenizing livers in 8 M urea containing 1% SDS using a polytron homogenizer and by centrifuging homogenates at 15,000 g for 10 min as previously described [13]. Proteins were separated with 10% SDS-PAGE and transferred to PVDF membrane. After blocking with 5% nonfat dry milk containing TBS-0.1% Tween20 buffer, membrane was probed with anti-CAR antibody (1∶5000, PP-N4111-00, Perseus, Tokyo, Japan), anti-HSP90 antibody (1∶1000, 610419, BD Transduction Laboratories, San Jose, CA), anti-TATA-binding protein (TBP) antibody (1∶2000, sc-273, Santa Cruz, Santa Cruz, CA), anti-βACTIN antibody (1∶2000, sc-47778, Santa Cruz), or anti-CCRP polyclonal antibody previously described [15] (1∶5000) in the blocking buffer for 2 h to overnight and 1 h with proper secondary HRP-conjugated mouse (sc-2314, 1∶5000) or rabbit antibody (sc-2004, 1∶5000). Protein bands on membrane were visualized using ECL prime detection reagent (GE healthcare, Buckinghamshire, UK). The bands were scanned and quantified using the ImageJ 1.47 software (National Institutes of Health, Bethesda, MD). Relative nuclear CAR levels were calculated by dividing the densities of CAR by those of TBP or βACTIN in each sample.

### cDNA Microarrays

Extracted RNAs obtained from livers using Trizol (Invitrogen, Carlsbad, CA) were purified with QIAGEN RNeasy kit (QIAGEN, Hilden, Germany) according to a manufacture's protocol. Gene expression analysis was conducted using Agilent Whole Mouse Genome 4×44 multiplex format oligo arrays (014868) (Agilent Technologies, Santa Clara, CA) following the Agilent 1-color microarray-based gene expression analysis protocol as reported previously [23]. In order to identify differentially expressed probes, analysis of variance (ANOVA) was used to determine if there was a statistical difference between the means of groups. Gene tags were identified as PB responsive genes if *p*-value was smaller than 0.05. To investigate functional characteristics as well as upstream regulators of specific gene lists, we used Ingenuity Pathway Analysis (IPA, Ingenuity Systems; www.ingenuity.com). cDNAs obtained from non-treated animals fasted for 24 h were also investigated by microarray analysis as described above. GEO accession number for our data is GSE56557.

### RT-PCR

Total RNAs were extracted from the livers of WT and KO males using Trizol. cDNAs were synthesized using SuperScript first strand synthesis system (Invitrogen) with random hexamers as primers. Real-time PCR was performed with an ABI Prism 7700 sequence detector (Applied Biosystems, Foster City, CA), TaqMan Universal PCR reaction mix and primers (Applied Biosystems) for mouse *Cyp2b10*, *Cyp2c55* or glyceraldehyde-3-phosphate dehydrogenase (*Gapdh*) as a control.

### Chromatin immunoprecipitation (ChIP)

After sacrifice by cervical dislocation, liver samples were collected and ChIP samples were prepared as reported previously [24]. ChIP assays were performed using ChIP-IT Express (Active Motif, Carlsbad, CA) according to the manufacturer's protocol with 10 µg of sheared chromatin and 2 µg of anti-human RXRα antibody (Santa Cruz, D-20X), anti-RNA polymerase II phospho Ser-5, an activated form (ab5131, Abcam, Cambridge, UK), anti-histone H3 lysine 27 trimethylation antibody (H3K27me3; 39155, Active Motif) or control rabbit IgG (2729, Cell Signaling, Danvers, MA). After purification of ChIP DNA samples with QIA PCR purification kit (QIAGEN), *Cyp2b10* enhancer region containing PBREM (−2440/−2238) and promoter region containing TATA box (-155/+69) were amplified by PCR using the following specific primers: 5′-GCTAATGCCTGTCTGGATCAGGA-3′ and 5′-GGAATACTGACCCAAGTTCAGTG-3′ (PBREM); 5′-AAGGGAATGAGGAGTGAGC-3′ and 5′-CAAGAAGCCCACAAGGAGAG-3′ (TATA). PCR reactions were performed with Green Taq PCR polymerase (Promega) at 94°C for 2 min followed by 30-35 cycles of 94°C for 30 s, 55°C for 30 s and 72°C for 30 s.

### Liver sections and staining

Following fixation of the livers with 10% formalin/phosphate-buffered saline, paraffin-embedded sections were subjected to standard Hematoxylin and Eosin (HE) staining. Hepatic lipid content was determined by 10 µm thick frozen sections stained with Oil Red O (Sigma–Aldrich, St. Louis, MO, USA).

### Serum cholesterol levels

After sacrifice by carbon-dioxide, blood was collected and serum levels of total cholesterol, HDL and LDL were determined as described previously [25].

### Statistical analysis

Values are plotted as the mean ± SE. Statistical analysis was conducted with GraphPad Prism 5 (GraphPad Software, San Diego, CA). ANOVA or unpaired *t*-test was used to determine significant differences among genotypes and treatments.

## Results

### CCRP KO mice

The CCRP global knockout mice were generated through genetic manipulations as shown in Fig. 1-A. Deletion of exon 2 to exon 4 is predicted to result in the splicing of exon 1 to exon 5. This results in an open reading frameshift, leading to a premature stop codon. Translation of the truncated mRNA results in an N-terminal peptide of just 38 amino acids residues (25 residues of CCRP followed by 13 missense residues). In these residues, important domains of CCRP for its function, 2 TPR domains and J-domain, are not included. Southern hybridization analysis confirmed the deletion of the *Ccrp* gene in the mouse genome (Fig. 1-B), whereas Western blot analysis using anti-mouse CCRP polyclonal antibody demonstrated the presence of CCRP at protein levels within both the cytoplasm and nucleus of WT but not in those of KO males (Fig. 1-C).

### Attenuated induction of CYP2B10 mRNA by PB

**Figure 2:**
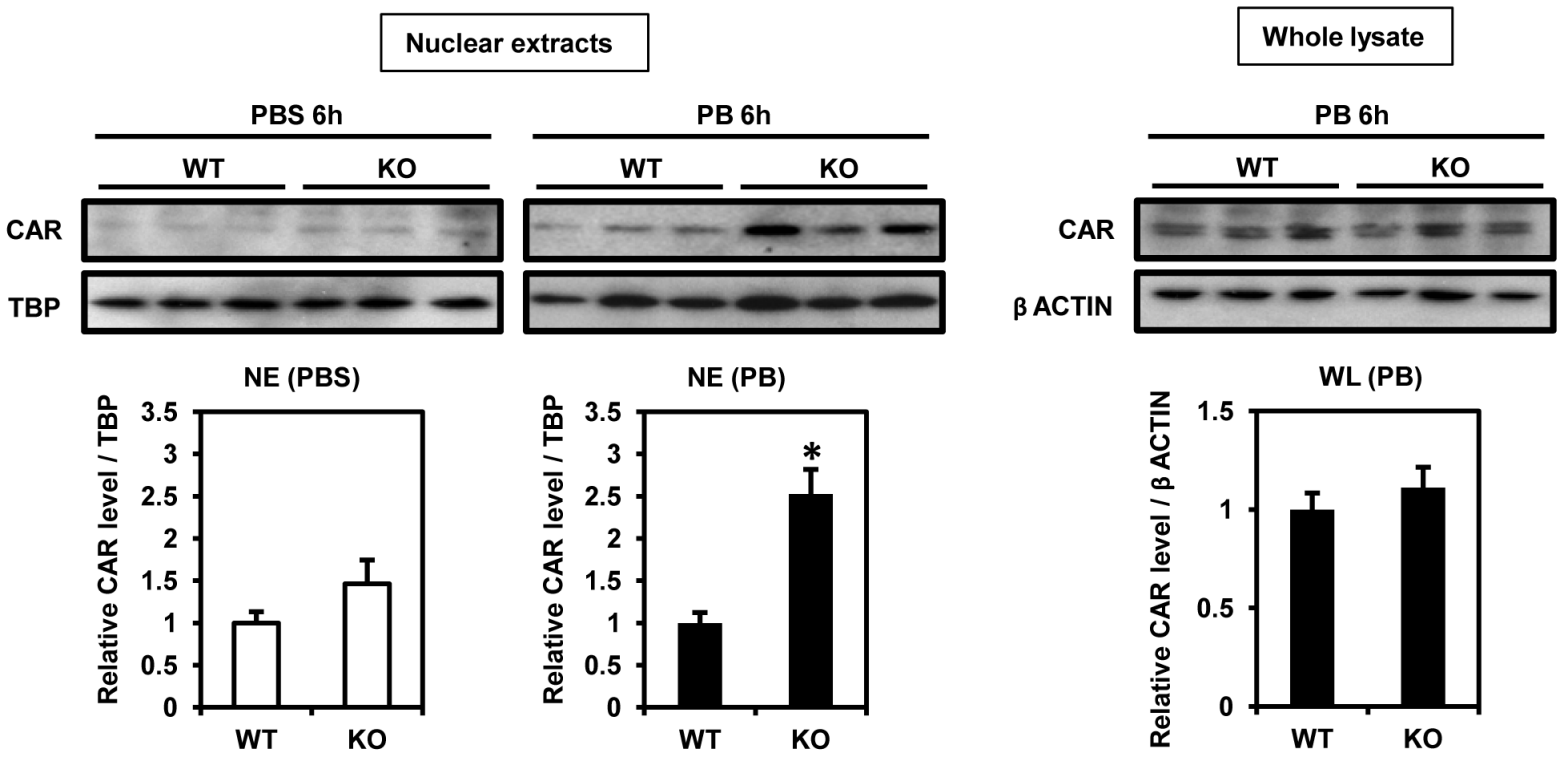
CAR protein levels after PB treatment. Fifteen microgram aliquots of liver nuclear extract or whole lysate were used for western blot analysis as described in materials and method section. Nuclear accumulation of CAR after 6 h PB treatment in WT and KO males on the left. On the right, total CAR protein levels in liver whole lysate prepared from PB-treated WT and KO males. We used TBP and β-Actin as loading control of nuclear extract and whole lysate, respectively. Columns denote mean ± SE determined in at 3 individual animals. Opened and closed columns represent control and PB-treated animals, respectively. Unpaired t-test was used to compare the relative CAR levels between genotypes (*WT vs KO, *p*<0.05). This experiment was repeated twice with different sample set prepared from different animals.

First, we checked the nuclear accumulation of CAR in livers of WT and KO males after PB treatment. As shown in Fig. 2, while CAR accumulated in the nuclear extracts from WT males after PB treatment, this accumulation was 2.5-fold greater in those from PB-treated KO males. In control animals, the levels of nuclear CAR were higher in the extracts from KO males compared with those from WT males but not statistically significant. As for total expression of CAR in liver, there was no difference between male PB-treated WT and KO mice (Fig. 2). These results suggest that CAR expressed in KO males was preferably localized in the nuclear compartment after PB treatment.

**Figure 3:**
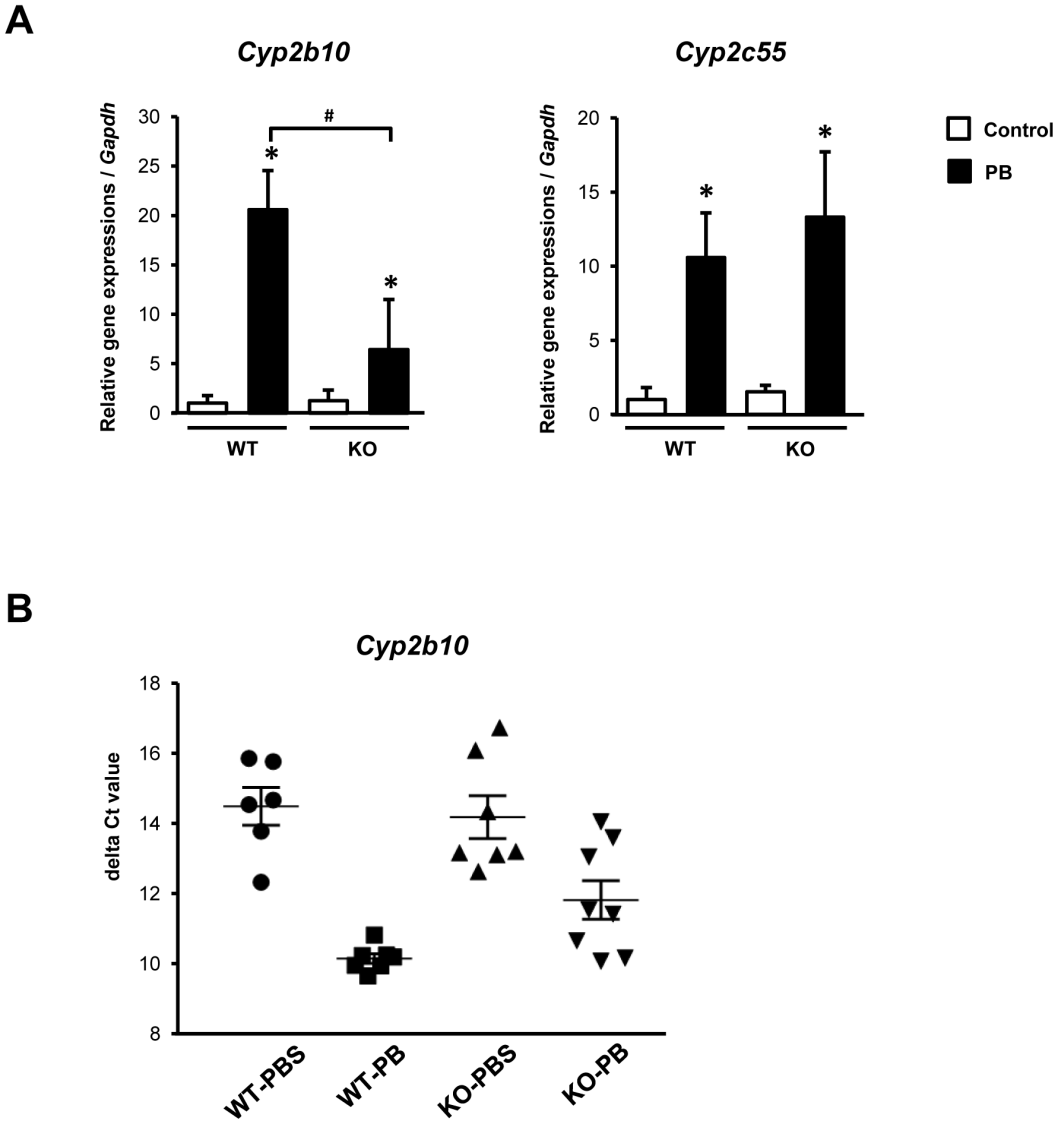
The mRNA expressions of CAR target genes in the livers after 6 h PB treatment. (A) CYP2B10 and CYP2C55 mRNA levels relative to GAPDH mRNA were measured by qRT-PCR. The relative levels were expressed as a ratio to that of PBS-treated WT mice. Columns denote mean ± SE determined in at 6-8 individual animals. Opened and closed columns represent control and PB-treated animals, respectively. One way-ANOVA was used to compare delta Ct values among groups (*control vs PB-treated, *p*<0.05; #WT vs KO, *p*<0.05). (B) Delta Ct values of CYP2B10 mRNA levels measured by qRT-PCR. The value was calculated by subtracting GAPDH Ct value from CYP2B10 Ct value in each animal.

cDNA microarray analysis was performed to examine the scope of gene expression in the livers of KO mice after PB treatment. Overview of the Top-10 most differentially up- or down-regulated genes is available in S1 Table. Numbers of genes which were either up- or down-regulated in the livers after PB treatment were 1302 and 2744 in WT and KO mice, respectively. Among CAR-regulated *Cyp* genes, PB-induction of CYP2B10 and 2B13 mRNAs was repressed about 5-fold in KO as compared to those in WT mice, while CYP2C39, CYP2C55 and CYP3A5 mRNAs were increased higher in PB-treated KO than WT mice (Table 1). Thus, CCRP appears to either attenuate or augment PB-induced activation depending on the types of *Cyp* genes. Real time PCR analysis of liver RNA samples confirmed a significant decrease in the induction rates of CYP2B10 mRNA in PB-treated KO males; only 5-fold in KO males as compared with 20-fold in WT males (Fig. 3-A). In the case of CYP2C55 mRNA, there was no significant difference in the induction ratio between WT and KO, consistent with microarray analysis. Here we focused our investigations on the *Cyp2b10* gene, the classic CAR target of PB induction. The similar attenuation of the induction of CYP2B10 mRNA was also observed in the induction in KO males after treatment with 1,4-bis[2-(3,5-dichloropyridyloxy)]benzene (TCPOBOP, a mouse CAR ligand) (data not shown). Noticeably, the induced levels of CYP2B10 mRNA exhibited relatively large variations from no to full induction in KO livers (Fig. 3-B), reasons for which will be investigated in the future. Nevertheless, CCRP appeared to be a critical factor for the optimal induction of the *Cyp2b10* gene.

**Table 1:** Up- and down regulation of CAR-regulated genes in the livers of WT and KO treated with PB.

			Fold change	
Reported	Gene symbol	Gene name	KO	WT
Up-regulated	*Ces2a*	*carboxylesterase 2 a*	2.493	2.038
	*Cyp2b13*	*cytochrome P450 family 2, subfamily b, polypeptide 13*	4.695	22.644
	*Cyp2b10*	*cytochrome P450 family 2, subfamily b, polypeptide 10*	5.536	28.008
	*Cyp2c39*	*cytochrome P450 family 2, subfamily c, polypeptide 39*	10.059	2.542
	*Cyp2c55*	*cytochrome P450 family 2, subfamily c, polypeptide 55*	6.237	4.995
	*Cyp3a44*	*cytochrome P450 family 3, subfamily a, polypeptide 44*	7.582	
	*Dio1*	*deiodinase, iodothyronine, type I*	3.210	2.387
	*Gadd45b*	*growth arrest and DNA-damage-inducible 45 beta*		3.844
	*Gsta2*	*glutathione S-transferase, alpha 2*	2.604	
	*Insig1*	*insulin induced gene 1*	2.946	
	*Insig2*	*insulin induced gene 2*		2.194
Down-regulated	*Cyp7a1*	*cytochrome P450, family 7, subfamily a, polypeptide 1*	3.289	

Blank means no significant alteration by PB. Cut-off: 2.0, *p*<0.05 (ANOVA).

### Chromatin-based regulation of the *Cyp2b10* promoter

**Figure 4:**
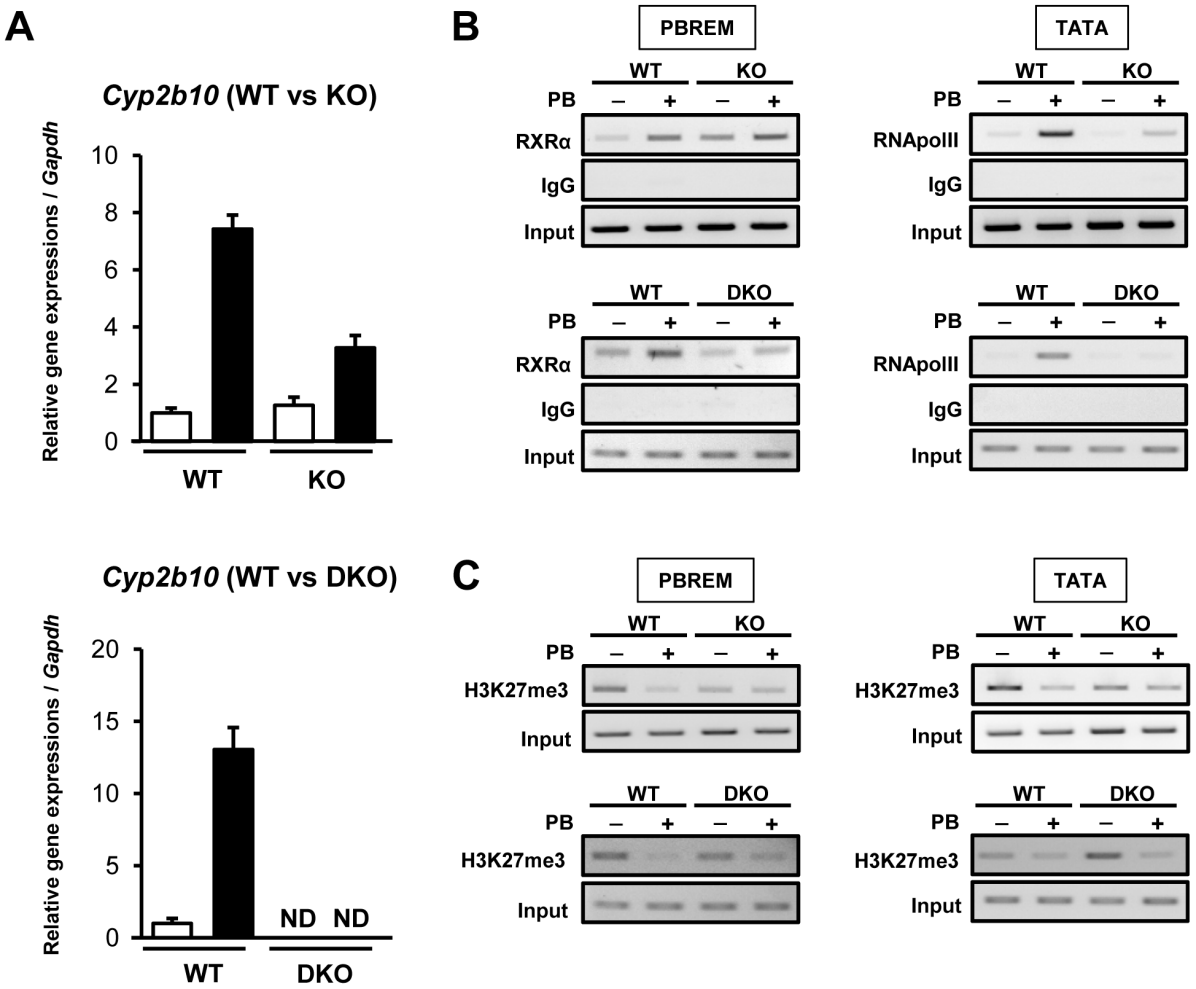
The recruitment of transcription factors and histone de-methylation after PB treatment. (A) The mRNA expressions of CYP2B10 in the livers of WT, KO and DKO mice after 3 h PB treatment. Columns denote mean ± SD determined in triplicates. Opened and closed columns represent control and PB-treated animals, respectively. (B) PB-induced recruitment of CAR/RXRα and RNA polymerase II in the *Cyp2b10* promoter in the livers of WT and KO; WT and DKO mouse liver. Precipitation of DNA fragments by anti-RXRα and anti-RNA polymerase II antibodies at PBREM and TATA box-containing region, respectively, was determined. (C) Histone de-methylation after PB treatment in the *Cyp2b10* promoter of WT and KO; WT and DKO mouse liver. Precipitation of DNA fragments by antibodies against the repressive mark H3K27me3. For both, precipitation of DNA fragments by normal IgG was used as negative control and DNA fragments without immunoprecipitation (Input) were used as positive control. Data shown are representative of results from two individual experiments.

ChIP assays were employed to examine the recruitment of CAR/RXRα complex to PBREM of the *Cyp2b10* promoter. Since existing CAR antibodies did not work for this assay, an anti-RXRα antibody was utilized as previously reported [24]. ChIP assay with PB-treated DKO mice confirmed that the increase of RXRα binding at PBREM is CAR-dependent. Consistent with results of qPCR, the CYP2B10 mRNA induction by PB in KO mouse liver was partially attenuated and completely abolished in DKO mouse liver, compared to WT (Fig. 4-A). These results confirmed that CAR is an essential factor for the *Cyp2b10* gene activation also in the absence of CCRP.

RXRα binding increased in the livers of WT males after PB treatment. This binding was already increased in KO males before PB treatment and further increased after treatment to the same levels as observed in PB-treated WT males (Fig. 4-B, upper left panel). No binding increases occurred in the livers of DKO males before or after PB treatment (Fig. 4-B, lower left panel). Thus, PB-induced increase in the CAR-mediated RXRα binding to PBREM did not appear to require the presence of CCRP. Next, binding of activated RNA polymerase II to a TATA box region of the *Cyp2b10* promoter was examined. ChIP assays revealed an increased binding of RNA polymerase II to TATA box in the livers of WT males after PB treatment, while this PB-induced increase was barely observable in those of either KO or DKO males (Fig. 4-B, right panels). Thus, CAR binding to PBREM resulted in the recruitment of activated RNA polymerase II in the presence of CCRP but not in its absence.

### Epigenetic regulation

In addition to promoter binding of CAR/RXRα or RNA polymerase II, we checked histone trimethylation levels in the promoter because histone H3 lysine 27 trimethylation (H3K27me3) level has been reported to decrease in response to PB treatment [26]. Trimethylation levels at H3K27 were greatly reduced by PB treatment within promoter regions which encode PBREM or TATA box only after PB treatment in the livers of WT males. On the other hand, these regions were already demethylated in the livers of KO males before PB treatment and no further de-methylation occurred after treatment (Fig. 4-C, upper panels). Contrary to what was observed with WT livers, the histone in the promoter remained methylated before treatment but demethylated after treatment in DKO livers (Fig. 4-C, lower panels). Thus, CAR binding allowed demethylation of the histone in the promoter without being activated in the absence of CCRP, while PB treatment demethylated it without activating the promoter in the absence of both CCRP and CAR.

### Steatotic livers and high cholesterol levels in serum

**Figure 5:**
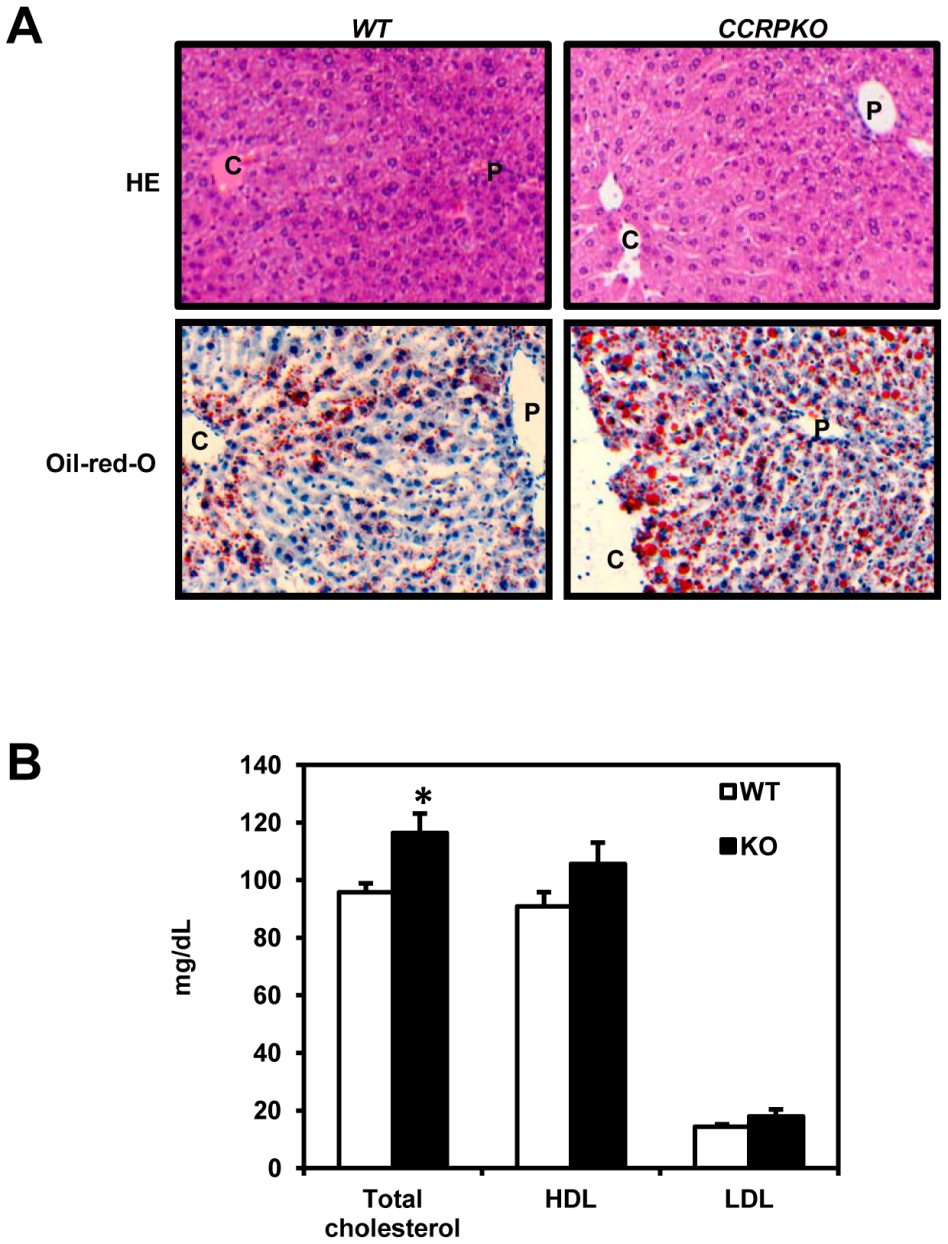
Development of steatotic liver in non-treated KO males. (A) HE or Oil-red-O staining of livers from WT and KO males fasted for 24 h. *C*: central vein; *P*: portal vein. Magnification, 200x. (B) Serum levels of total cholesterol, HDL and LDL in males. Columns denote mean ± SE determined in 6 individual animals. Opened and closed columns represent WT and KO males, respectively. Unpaired t-test was used to compare the levels between genotypes (*WT vs KO, *p*<0.05).

KO males were found to develop steatotic livers in response to 24 h fasting. Fig. 5-A shows microscopic images of WT and KO liver sections after 24 h starvation stained by HE or Oil-red-O. The increase of Oil-red-O stained vesicles suggested higher lipid accumulation in KO mouse livers particularly, around central veins (Fig. 5-A). On the other hand, the lack of CCRP did not affect serum triglyceride levels (105.3 mg/dL in WT; 104.5 mg/dL in KO).

Serum cholesterol concentrations were significantly higher in KO males, as compared to those in WT (116.3 mg/dL in KO and 95.8 mg/dL in WT). Serum levels of HDL and LDL were also increased in KO males (HDL, 105.7 and 90.8 mg/dL in KO and WT, respectively; LDL, 18.0 and 14.3 mg/dL in KO and WT, respectively). However, these differences in the HDL and LDL levels were not statistically significant.

cDNA microarray analysis suggested the activation of cholesterol metabolism in KO mouse liver (Table 2) and revealed an increase of mRNAs of enzymes and factors that are involved in cholesterol biosynthesis such as *Cyp51A1*, *Hmgcs1* (3-hydroxy-3-methylglutaryl-CoA synthase 1) and *Sqle* (squalene epoxydase) (Table 3). Upstream analysis with IPA predicted the activation of sterol regulatory element-binding protein 1 and 2 (SREBP1 and 2) and SREBP-cleavage-activating protein (SCAP) with the activation z-scores of 4.653, 3.873 and 4.418 for SREBP1, SREBP 2 and SCAP, respectively.

**Table 2:** Top 5 canonical pathways (WT v.s. KO after 24 h fasting).

Ingenuity Canonical Pathways	p-value	Ratio
Superpathway of Cholesterol Biosynthesis	7.48E-05	11/27
Glutathione-mediated Detoxification	4.87E-04	9/23
Cholesterol Biosynthesis I	1.61E-03	6/13
Cholesterol Biosynthesis II (via 24,25-dihydrolanosterol)	1.61E-03	6/13
Cholesterol Biosynthesis III (via Desmosterol)	1.61E-03	6/13

IPA suggested these pathways are significantly altered by CCRP KO in male mouse liver (ANOVA, *p*<0.05). Ratio indicates the number of genes significantly altered within genes related to each pathway.

**Table 3:** Cholesterol biosynthesis gene expressions after 24 h fasting.

gene		Fold change
*Acat3*	*acetyl-Coenzyme A acetyltransferase 3*	1.817
*Cyp51a*	*cytochrome P450, family 51, subfamily a*	2.326
*Fdps*	*farnesyl diphosphate synthase*	1.818
*Hmgcr*	*3-hydroxy-3-methylglutaryl-CoA reductase*	1.898
*Hmgcs1*	*3-hydroxy-3-methylglutaryl-CoA synthase 1 (soluble)*	2.266
*Idi1*	*isopentenyl-diphosphate delta isomerase 1*	2.714
*Msmo1*	*methylsterol monooxygenase 1*	1.980
*Nsdhl*	*NAD(P) dependent steroid dehydrogenase-like*	1.965
*Sqle*	*squalene epoxidase*	3.677

Listed genes were significantly higher in KO, compared to WT. Cut-off: 1.5, *p*<0.05 (ANOVA).

## Discussion

Our previous study suggested that CCRP directly binds to CAR and accumulates it in the cytoplasm of HepG2 cells [15]. Our present work with livers of CCRP KO mice has now confirmed that CCRP regulates CAR-mediated activation of the *Cyp2b10* gene as well as the intracellular localization of CAR.

Since CCRP is present in both cytoplasm and nucleus in the livers, CCRP can regulate CAR activity in either or both compartments. Co-chaperone regulation in the cytoplasm has been intensively investigated in nuclear receptors. For example: FK506 binding protein 51 (FKBP51) and 52, TPR proteins within the immunophilin family, mediate the interaction between GR with HSP90 to facilitate ligand binding. Liganded GR replaces FKBP51 with FKBP52 to translocate into the nucleus [27], [28]. This role of FKBP52 was confirmed in a cell line derived from FKBP52 KO mice, although the corresponding GR-FKBP52 complex could not be found in the cytoplasm of rat livers [29], [30]. Hepatitis B virus X-associated protein 2 (XAP2), also known as AIP or ARA9, the other immunophilin type of TPR protein, promotes interaction between aryl hydrocarbon receptor (AHR) and HSP90 to translocate AHR from the cytoplasm into the nucleus after ligand binding in transformed cells such as Hepa1 cells [31]–[34]. Global knock out of XAP2 was embryonic lethal [35]. Liver-specific XAP2 KO mouse was produced [36]; however, neither an AHR-XAP2 complex nor intracellular localization has been confirmed before or after ligand treatments in the livers. Global CCRP KO mice grow normally and as to CCRP in the livers *in vivo*, it appears to constitute a regulatory system that optimizes nuclear CAR accumulation by its ability of repressing this accumulation. In addition to CAR, CCRP also interacted with GR, mineralocorticoid receptor, progesterone receptor (PR), estrogen receptor, androgen receptor and pregnane X receptor (PXR) [20], [37]–[39]. CCRP regulates interactions between PR and HSP90, and GR and HSP70 [20], [38]. In the cases of PXR, over-expression of CCRP increased the cytoplasmic level of PXR-CCRP-HSP90 complex and retained it in the cytoplasm of HepG2 cell [37]. Besides nuclear receptors, CCRP also interacted with p53 to inhibit its interaction with mouse double minute 2 homologue (MDM2) in COS1 cells [40]. Therefore, CCRP may be a common co-chaperone and the roles it plays in the cytoplasm may go far beyond CAR to many other nuclear receptors as well as signaling molecules.

CCRP is now found to engage in diverse regulations in the nucleus, one of which is epigenetic regulation. Only in the presence of CCRP does the histone of *Cyp2b10* promoter remain methylated before PB treatment and demethylated after treatment. It appears that only when this epigenetic regulation is properly integrated into CAR function that the demethylated promoter recruits RNA polymerase II for activation. On the other hand, aberrant demethylation, which occurs in the absence of CCRP or both CCRP and CAR does not enable the promoter to recruit RNA polymerase II. According to Hesterman and Brown, the AHR antagonist 3,3′-diindolylmethane (DIM) can induce AHR recruitment to the *Cyp1a1* promoter but cannot activate the gene because of the failure of histone acetylation and the recruitment of RNA polymerase II [41]. Like what different ligands can do, CCRP may determine how CAR recruits co-activators an RNA polymerase II to the promoter.

There is another possibility that explains the attenuation of *Cyp2b10* gene activation in CCRP KO mice: CCRP may be directly utilized as a transcriptional co-activator in the nucleus. There is an example that co-chaperones can work as transcriptional co-regulators. Hjd1/DNAJB1, which belongs to the HSP40 family, acts as a transcriptional repressor to down-regulate heat shock-induced factor 1 (HSF1)-mediated gene activation because HSP70 and the co-chaperone DNAJB1 interact directly with the transactivation domain of HSF1 and over-expression of them represses heat shock gene transcription [42]. ChIP assays were performed with either our own or a commercially available CCRP antibody but neither antibody was suitable for these assays.

CCRP co-regulated many of CAR-regulated *Cyp* genes which are also known to be regulated by other nuclear receptors. For example, HNF4α has been reported to regulate *Cyp2b10*, *Cyp2b13*, and *Cyp3a44* [43]. HNF4α mRNA remained unchanged in the liver by CCRP KO and down-regulated only slightly after PB treatment (data not shown). However, the interactions between CCRP and HNF4α have not been investigated, thereby remaining the possibility that HNF4α is involved in the co-regulation by CCRP. On the other hand, this type of co-chaperone-mediated specificity was previously demonstrated within AHR-regulated *CYP* genes, the *Cyp1b1* gene required the co-chaperone XAP for its full induction by TCDD, but XAP was dispensable for the full induction of the *Cyp1a1* and *Cyp1a2* genes [36]. These observations suggest that co-regulations by co-chaperones can be gene-specific; biological meanings of this specificity and its molecular mechanisms should be interesting subjects for future investigations.

Our histochemical staining of liver section with HE or oil red revealed development of steatosis and accumulation of neutral lipids in livers of CCRP KO males after 24 h fasting while no clear difference was observed in fed animals between WT and KO (data not shown). Consistent with this result, microarray analysis showed that cholesterol biosynthesizing genes were up-regulated in the livers of KO males such as *Cyp51A1*, *Hmgcs1* (3-hydroxy-3-methylglutaryl-CoA synthase 1) and *Sqle* (squalene epoxydase) (Tables 2 and 3). Other than *Acat3* (acetyl-Coenzyme A acetyltransferase 3), cholesterol synthesizing genes in Table 3 are transcriptionally regulated by SREBP1/2. Upstream analysis with IPA strongly suggested the activation of SREBP in the liver of CCRP KO mice. In addition to SREBP, since many of these genes are known to be regulated through CAR and/or PXR [44], [25], CCRP could regulate them either independently or by co-regulating nuclear receptors.

In conclusion, CCRP is capable of regulating CAR activities in both cytoplasm and nucleus in the livers *in vivo*. CCRP, acting as a co-activator, enables CAR for the *Cyp2b10* promoter to recruit RNA polymerase II. This co-regulation by CCRP may expand to other genes in future investigations. Hepatic steatosis and an increase in blood cholesterol levels suggest that CCRP may regulate diverse array of hepatic genes far beyond *Cyp* genes. Further investigations with KO mice should help us to understand CCRP biology and its molecular mechanisms.

## Supporting Information

S1 Table **Overview of top-10 up- and down-regulated genes after PB (** ***p*** **<0.05, ANOVA).** Genes represented in capital are human homolog.
(DOCX)

## References

[1] Honkakoski P, Negishi M (1998) Regulatory DNA elements of phenobarbital-responsive cytochrome P450 CYP2B genes. J Biochem Mol Toxicol 12:3–9. 9414482 10.1002/(sici)1099-0461(1998)12:1<3::aid-jbt2>3.0.co;2-p

[2] Sueyoshi T, Kawamoto T, Zelko I, Honkakoski P, Negishi M (1999) The repressed nuclear receptor CAR responds to phenobarbital in activating the human CYP2B6 gene. J Biol Chem 274:6043–6046. 10037683 10.1074/jbc.274.10.6043

[3] Wei P, Zhang J, Egan-Hafley M, Liang S, Moore DD (2000) The nuclear receptor CAR mediates specific xenobiotic induction of drug metabolism. Nature 407:920–923. 11057673 10.1038/35038112

[4] Sueyoshi T, Negishi M (2001) Phenobarbital response elements of cytochrome P450 genes and nuclear receptors. Annu Rev Pharmacol Toxicol 41:123–143. 11264453 10.1146/annurev.pharmtox.41.1.123

[5] Sugatani J, Yamakawa K, Yoshinari K, Machida T, Takagi H, et al (2002) Identification of a defect in the UGT1A1 gene promoter and its association with hyperbilirubinemia. Biochem Biophys Res Commun 292:492–497. 11906189 10.1006/bbrc.2002.6683

[6] Ueda A, Hamadeh HK, Webb HK, Yamamoto Y, Sueyoshi T, et al (2002) Diverse roles of the nuclear orphan receptor CAR in regulating hepatic genes in response to phenobarbital. Mol Pharmacol 61:1–6. 11752199 10.1124/mol.61.1.1

[7] Kakizaki S, Yamamoto Y, Ueda A, Moore R, Sueyoshi T, et al (2003) Phenobarbital induction of drug/steroid-metabolizing enzymes and nuclear receptor CAR. Biochim Biophys Acta 1619:239–242. 12573483 10.1016/s0304-4165(02)00482-8

[8] Yamamoto Y, Moore R, Goldsworthy TL, Negishi M, Maronpot RR (2004) The orphan nuclear receptor constitutive active/androstane receptor is essential for liver tumor promotion by phenobarbital in mice. Cancer Res 64:7197–7200. 15492232 10.1158/0008-5472.CAN-04-1459

[9] Phillips JM, Yamamoto Y, Negishi M, Maronpot RR, Goodman JI (2007) Orphan nuclear receptor constitutive active/androstane receptor-mediated alterations in DNA methylation during phenobarbital promotion of liver tumorigenesis. Toxicol Sci 96:72–82. 17172636 10.1093/toxsci/kfl188

[10] Kawamoto T, Sueyoshi T, Zelko I, Moore R, Washburn K, et al (1999) Phenobarbital-responsive nuclear translocation of the receptor CAR in induction of the CYP2B gene. Mol Cell Biol 19:6318–6322. 10454578 10.1128/mcb.19.9.6318 PMC84602

[11] Mutoh S, Osabe M, Inoue K, Moore R, Pedersen L, et al (2009) Dephosphorylation of threonine 38 is required for nuclear translocation and activation of human xenobiotic receptor CAR (NR1I3). J Biol Chem 284:34785–34792. 19858220 10.1074/jbc.M109.048108 PMC2787341

[12] Koike C, Moore R, Negishi M (2007) Extracellular signal-regulated kinase is an endogenous signal retaining the nuclear constitutive active/androstane receptor (CAR) in the cytoplasm of mouse primary hepatocytes. Mol Pharmacol 71:1217–1221. 17314319 10.1124/mol.107.034538 PMC2100393

[13] Mutoh S, Sobhany M, Moore R, Perera L, Pedersen L, et al (2013) Phenobarbital indirectly activates the constitutive active androstane receptor (CAR) by inhibition of epidermal growth factor receptor signaling. Sci Signal 6:ra31. 23652203 10.1126/scisignal.2003705 PMC5573139

[14] Yang H, Garzel B, Heyward S, Moeller T, Shapiro P, et al (2014) Metformin Represses Drug-Induced Expression of CYP2B6 by Modulating the Constitutive Androstane Receptor Signaling. Mol Pharmacol 85:249–260. 24252946 10.1124/mol.113.089763 PMC3913356

[15] Kobayashi K, Sueyoshi T, Inoue K, Moore R, Negishi M (2003) Cytoplasmic accumulation of the nuclear receptor CAR by a tetratricopeptide repeat protein in HepG2 cells. Mol Pharmacol 64:1069–1075. 14573755 10.1124/mol.64.5.1069

[16] Bukau B, Horwich AL (1998) The Hsp70 and Hsp60 chaperone machines. Cell 92:351–366. 9476895 10.1016/s0092-8674(00)80928-9

[17] Greene MK, Maskos K, Landry SJ (1998) Role of the J-domain in the cooperation of Hsp40 with Hsp70. Proc Natl Acad Sci U S A 95:6108–6113. 9600925 10.1073/pnas.95.11.6108 PMC27593

[18] Lamb JR, Tugendreich S, Hieter P (1995) Tetratrico peptide repeat interactions: to TPR or not to TPR? Trends Biochem Sci 20:257–259. 7667876 10.1016/s0968-0004(00)89037-4

[19] Scheufler C, Brinker A, Bourenkov G, Pegoraro S, Moroder L, et al (2000) Structure of TPR domain-peptide complexes: critical elements in the assembly of the Hsp70-Hsp90 multichaperone machine. Cell 101:199–210. 10786835 10.1016/S0092-8674(00)80830-2

[20] Brychzy A, Rein T, Winklhofer KF, Hartl FU, Young JC, et al (2003) Cofactor Tpr2 combines two TPR domains and a J domain to regulate the Hsp70/Hsp90 chaperone system. EMBO J 22:3613–3623. 12853476 10.1093/emboj/cdg362 PMC165632

[21] Inagaki M, Komatsu Y, Scott G, Yamada G, Ray M, et al (2008) Generation of a conditional mutant allele for Tab1 in mouse. Genesis 46:431–439. 18693278 10.1002/dvg.20418 PMC2637350

[22] Sueyoshi T, Kobayashi R, Nishio K, Aida K, Moore R, et al (1995) A nuclear factor (NF2d9) that binds to the male-specific P450 (Cyp 2d-9) gene in mouse liver. Mol Cell Biol 15:4158–4166. 7623810 10.1128/mcb.15.8.4158 PMC230654

[23] Saito K, Negishi M, James Squires E (2013) Sexual dimorphisms in zonal gene expression in mouse liver. Biochem Biophys Res Commun 436:730–735. 23791742 10.1016/j.bbrc.2013.06.025

[24] Saito K, Moore R, Negishi M (2013) Nuclear receptor CAR specifically activates the two-pore K+ channel Kcnk1 gene in male mouse livers, which attenuates phenobarbital-induced hepatic hyperplasia. Toxicol Sci 132:151–161. 23291559 10.1093/toxsci/kfs338 PMC3576010

[25] Nakamura K, Moore R, Negishi M, Sueyoshi T (2007) Nuclear pregnane X receptor cross-talk with FoxA2 to mediate drug-induced regulation of lipid metabolism in fasting mouse liver. J Biol Chem 282:9768–9776. 17267396 10.1074/jbc.M610072200 PMC2258557

[26] Lempiäinen H, Müller A, Brasa S, Teo SS, Roloff TC, et al (2011) Phenobarbital mediates an epigenetic switch at the constitutive androstane receptor (CAR) target gene Cyp2b10 in the liver of B6C3F1 mice. PLoS One 6:e18216. 21455306 10.1371/journal.pone.0018216 PMC3063791

[27] Echeverría PC, Mazaira G, Erlejman A, Gomez-Sanchez C, Piwien Pilipuk G, et al (2009) Nuclear import of the glucocorticoid receptor-hsp90 complex through the nuclear pore complex is mediated by its interaction with Nup62 and importin beta. Mol Cell Biol 29:4788–4797. 19581287 10.1128/MCB.00649-09 PMC2725705

[28] Riggs DL, Roberts PJ, Chirillo SC, Cheung-Flynn J, Prapapanich V, et al (2003) The Hsp90-binding peptidylprolyl isomerase FKBP52 potentiates glucocorticoid signaling in vivo. EMBO J 22:1158–1167. 12606580 10.1093/emboj/cdg108 PMC150341

[29] Hedman E, Widén C, Asadi A, Dinnetz I, Schröder WP, et al (2006) Proteomic identification of glucocorticoid receptor interacting proteins. Proteomics 6:3114–3126. 16619302 10.1002/pmic.200500266

[30] Warrier M, Hinds TD, Ledford KJ, Cash HA, Patel PR, et al (2010) Susceptibility to diet-induced hepatic steatosis and glucocorticoid resistance in FK506-binding protein 52-deficient mice. Endocrinology 151:3225–3236. 20427484 10.1210/en.2009-1158 PMC2903936

[31] Meyer BK, Pray-Grant MG, Vanden Heuvel JP, Perdew GH (1998) Hepatitis B virus X-associated protein 2 is a subunit of the unliganded aryl hydrocarbon receptor core complex and exhibits transcriptional enhancer activity. Mol Cell Biol 18:978–988. 9447995 10.1128/mcb.18.2.978 PMC108810

[32] Meyer BK, Perdew GH (1999) Characterization of the AhR-hsp90-XAP2 core complex and the role of the immunophilin-related protein XAP2 in AhR stabilization. Biochemistry 38:8907–8917. 10413464 10.1021/bi982223w

[33] Petrulis JR, Hord NG, Perdew GH (2000) Subcellular localization of the aryl hydrocarbon receptor is modulated by the immunophilin homolog hepatitis B virus X-associated protein 2. J Biol Chem 275:37448–37453. 10986286 10.1074/jbc.M006873200

[34] Bell DR, Poland A (2000) Binding of aryl hydrocarbon receptor (AhR) to AhR-interacting protein. The role of hsp90. J Biol Chem 275:36407–36414. 10961990 10.1074/jbc.M004236200

[35] Lin BC, Sullivan R, Lee Y, Moran S, Glover E, et al (2007) Deletion of the aryl hydrocarbon receptor-associated protein 9 leads to cardiac malformation and embryonic lethality. J Biol Chem 282:35924–35932. 17916558 10.1074/jbc.M705471200

[36] Nukaya M, Lin BC, Glover E, Moran SM, Kennedy GD, et al (2010) The aryl hydrocarbon receptor-interacting protein (AIP) is required for dioxin-induced hepatotoxicity but not for the induction of the Cyp1a1 and Cyp1a2 genes. J Biol Chem 285:35599–35605. 20829355 10.1074/jbc.M110.132043 PMC2975184

[37] Squires EJ, Sueyoshi T, Negishi M (2004) Cytoplasmic localization of pregnane X receptor and ligand-dependent nuclear translocation in mouse liver. J Biol Chem 279:49307–49314. 15347657 10.1074/jbc.M407281200

[38] Moffatt NS, Bruinsma E, Uhl C, Obermann WM, Toft D (2008) Role of the cochaperone Tpr2 in Hsp90 chaperoning. Biochemistry 47:8203–8213. 18620420 10.1021/bi800770g

[39] Schülke JP, Wochnik GM, Lang-Rollin I, Gassen NC, Knapp RT, et al (2010) Differential impact of tetratricopeptide repeat proteins on the steroid hormone receptors. PLoS One 5:e11717. 20661446 10.1371/journal.pone.0011717 PMC2908686

[40] Kubo N, Wu D, Yoshihara Y, Sang M, Nakagawara A, et al (2013) Co-chaperon DnaJC7/TPR2 enhances p53 stability and activity through blocking the complex formation between p53 and MDM2. Biochem Biophys Res Commun 430:1034–1039. 23261415 10.1016/j.bbrc.2012.11.121

[41] Hestermann EV, Brown M (2003) Agonist and chemopreventative ligands induce differential transcriptional cofactor recruitment by aryl hydrocarbon receptor. Mol Cell Biol 23:7920–7925. 14560034 10.1128/MCB.23.21.7920-7925.2003 PMC207605

[42] Shi Y, Mosser DD, Morimoto RI (1998) Molecular chaperones as HSF1-specific transcriptional repressors. Genes Dev 12:654–666. 9499401 10.1101/gad.12.5.654 PMC316571

[43] Wiwi CA, Gupte M, Waxman DJ (2004) Sexually dimorphic P450 gene expression in liver-specific hepatocyte nuclear factor 4alpha-deficient mice. Mol Endocrinol 18:1975–1987. 15155787 10.1210/me.2004-0129

[44] Yamamoto Y, Kawamoto T, Negishi M (2003) The role of the nuclear receptor CAR as a coordinate regulator of hepatic gene expression in defense against chemical toxicity. Arch Biochem Biophys 409:207–211. 12464260 10.1016/s0003-9861(02)00456-3

