# Supplementary material for: The Roles of Co-Chaperone CCRP/DNAJC7 in Cyp2b10 Gene Activation and Steatosis Development in Mouse Livers
Source: PLoS One. 2014 Dec 26;9(12):e115663. doi: 10.1371/journal.pone.0115663 (PMC4277317; doi:10.1371/journal.pone.0115663)
Supplement: S1 Table — Overview of top-10 up- and down-regulated genes after PB ( p <0.05, ANOVA). Genes represented in capital are human homolog. (DOCX) [file pone.0115663.s001.docx]

Table S1. Overview of top-10 up- and down-regulated genes after PB (*p* < 0.05, ANOVA). Genes represented in capital are human homolog.

|  |  |  | Fold change | |
| --- | --- | --- | --- | --- |
|  | Agilent ID | gene | *WT* | *KO* |
| **Induced only in WT** | A_52_P251403 | *TGFBR2* | 9.491 | ― |
|  | A_51_P274803 | *Trim30b* | 8.966 | ― |
|  | A_51_P146223 | *KRTDAP* | 6.733 | ― |
|  | A_51_P343689 | *SLC14A1* | 6.681 | ― |
|  | A_52_P3698 | *G2E3* | 6.448 | ― |
|  | A_52_P579623 | *1700030N03Rik* | 6.265 | ― |
|  | A_52_P616659 | *LRCH2* | 6.136 | ― |
|  | A_51_P425401 | *KIF27* | 5.970 | ― |
|  | A_51_P331429 | *COLGALT2* | 5.884 | ― |
|  | A_52_P40569 | *1700066J03Rik* | 5.705 | ― |
|  |  |  |  |  |
| **Induced both in WT and KO** | A_52_P300533 | *2310043M15Rik* | 64.846 | 11.682 |
|  | A_52_P472486 | *Cyp2b10* | 28.008 | 5.536 |
|  | A_51_P492339 | *Cyp2b13* | 22.644 | 4.695 |
|  | A_52_P382149 | *CYP26A1* | 19.531 | 6.117 |
|  | A_52_P532982 | *GDF15* | 19.155 | 7.154 |
|  | A_51_P296608 | *GADD45A* | 16.544 | 19.378 |
|  | A_51_P259186 | *PPP1R42* | 4.730 | 15.356 |
|  | A_51_P221256 | *SLC34A2* | 4.888 | 10.344 |
|  | A_51_P304109 | *Cyp2c39* | 2.542 | 10.059 |
|  | A_51_P133562 | *SERPINA6* | 4.192 | 8.620 |
|  |  |  |  |  |
| **Induced only in KO** | A_51_P431539 | *PRSS56* | ― | 57.740 |
|  | A_52_P639461 | *CA3* | ― | 26.281 |
|  | A_51_P277683 | *MS4A1* | ― | 16.955 |
|  | A_51_P416771 | *RANBP3L* | ― | 14.254 |
|  | A_51_P244234 | *Gm20172* | ― | 13.977 |
|  | A_52_P661942 | *OR51M1* | ― | 12.560 |
|  | A_52_P610015 | *E130006D01Rik* | ― | 11.313 |
|  | A_52_P195602 | *CPLX2* | ― | 11.234 |
|  | A_51_P252126 | *Sry* | ― | 10.702 |
|  | A_52_P177635 | *SLC6A15* | ― | 9.790 |
|  |  |  |  |  |
| **Repressed only in WT** | A_52_P272429 | *EHF* | -9.454 | ― |
|  | A_52_P183421 | *CRAMP1L* | -9.329 | ― |
|  | A_51_P453446 | *ROR1* | -8.441 | ― |
|  | A_52_P489068 | *D330013E07Rik* | -7.990 | ― |
|  | A_51_P461894 | *TNNC1* | -6.997 | ― |
|  | A_51_P322465 | *IMP4* | -6.806 | ― |
|  | A_52_P456392 | *LIMCH1* | -6.707 | ― |
|  | A_51_P243559 | *4833431D13Rik* | -6.099 | ― |
|  | A_51_P521010 | *PPP1R3C* | -6.008 | ― |
|  | A_52_P460570 | *NXF2/NXF2B* | -5.723 | ― |
|  |  |  |  |  |
| **Repressed both in WT and KO** | A_52_P531610 | *AGXT2L1* | -19.087 | -16.014 |
|  | A_52_P87900 | *FAM107A* | -14.226 | -30.317 |
|  | A_51_P315904 | *GADD45G* | -12.297 | -15.820 |
|  | A_52_P37991 | *HOMER1* | -10.086 | -6.632 |
|  | A_52_P251623 | *FAM222A* | -6.805 | -9.979 |
|  | A_51_P164998 | *STMN2* | -11.064 | -37.899 |
|  | A_52_P87900 | *FAM107A* | -14.226 | -30.317 |
|  | A_51_P412955 | *PLCB4* | -4.112 | -24.624 |
|  | A_52_P483104 | *LPIN1* | -8.362 | -24.033 |
|  | A_51_P367866 | *EGR1* | -2.937 | -16.044 |
|  |  |  |  |  |
| **Repressed only in KO** | A_51_P155873 | *PPP1R3G* | ― | -104.308 |
|  | A_51_P434567 | *ADRA2A* | ― | -89.935 |
|  | A_51_P323518 | *CEND1* | ― | -40.367 |
|  | A_51_P245796 | *DDIT4* | ― | -31.008 |
|  | A_51_P114314 | *DUSP8* | ― | -27.134 |
|  | A_52_P423795 | *Gm9777* | ― | -24.220 |
|  | A_52_P140005 | *NIPAL1* | ― | -20.641 |
|  | A_51_P286018 | *CDC42EP5* | ― | -19.796 |
|  | A_51_P428360 | *Retnlb* | ― | -19.543 |
|  | A_52_P50529 | *NLRP12* | ― | -15.968 |
